# Optimization of Anodic Porous Alumina Fabricated from Commercial Aluminum Food Foils: A Statistical Approach

**DOI:** 10.3390/ma10040417

**Published:** 2017-04-15

**Authors:** Eva Riccomagno, Amirreza Shayganpour, Marco Salerno

**Affiliations:** 1 Department of Mathematics, University of Genova, via Dodecaneso 35, I-16146 Genova, Italy; riccomag@dima.unige.it; 2 Department of Nanophysics, Istituto Italiano di Tecnologia, via Morego 30, I-16163 Genova, Italy; amirreza.shayganopur@iit.it

**Keywords:** nanoporous materials, alumina, anodization, design of experiments, image analysis

## Abstract

Anodic porous alumina is a known material based on an old industry, yet with emerging applications in nanoscience and nanotechnology. This is promising, but the nanostructured alumina should be fabricated from inexpensive raw material. We fabricated porous alumina from commercial aluminum food plate in 0.4 M aqueous phosphoric acid, aiming to design an effective manufacturing protocol for the material used as nanoporous filler in dental restorative composites, an application demonstrated previously by our group. We identified the critical input parameters of anodization voltage, bath temperature and anodization time, and the main output parameters of pore diameter, pore spacing and oxide thickness. Scanning electron microscopy and grain analysis allowed us to assess the nanostructured material, and the statistical design of experiments was used to optimize its fabrication. We analyzed a preliminary dataset, designed a second dataset aimed at clarifying the correlations between input and output parameters, and ran a confirmation dataset. Anodization conditions close to 125 V, 20 °C, and 7 h were identified as the best for obtaining, in the shortest possible time, pore diameters and spacing of 100–150 nm and 150–275 nm respectively, and thickness of 6–8 µm, which are desirable for the selected application according to previously published results. Our analysis confirmed the linear dependence of pore size on anodization voltage and of thickness on anodization time. The importance of proper control on the experiment was highlighted, since batch effects emerge when the experimental conditions are not exactly reproduced.

## 1. Introduction

Anodic porous alumina (APA) is a nanostructured material obtained with good uniformity on large area scale (order of 10 cm × 10 cm, extendible to 1 m × 1 m) by means of inexpensive manufacturing process of anodization [1,2]. Anodization of aluminum (Al) has been carried out for almost a century [3], mainly for protective purposes against raw metal corrosion [4], as well as for decoration of the resulting surface when loading the oxide pores with a dye before sealing them on the very top. However, in the last 30 years APA fabrication has attracted the interest of nanotechnologists, interested in understanding the pores origin and growth processes [1,5,6]. The effects of different electrolytes [1,3] and their mixtures [7], as well as of viscosity [8] and additives [9,10], have also been extensively investigated. Important APA applications may be found in advanced optoelectronics [11,12], such as distributed feedback laser cavities or photonic crystals [13,14] and surface-enhanced Raman scattering substrate [15,16], as well as biomaterial for cell culture investigation [17,18] and surface coating of biomedical implants [19,20,21]. A great deal of work on chemical sensors and biosensors based on optical principles different from SERS has also been carried out [22], including drug delivery application on possible biomedical devices [23,24,25]. This condition of having a wide-spread old-standing industry and know-how—that of Al anodization—at service for a nanostructured material with increasing understanding and emerging applications in nanotechnology, is promising. However, except few cases [26], most of the nanotechnology studies mentioned above have been carried out on ultrapure, laboratory-specification raw material. This approach is expensive and prevents the use of this simple, large-area fabrication technique of anodization at the production scale. In order to open the way for applications of APA in—possibly disposable—devices and/or products with broad spread on the market, use of much inexpensive raw material should be adopted. This work presents the results of a statistically designed experimental study carried out on anodization of a consumer product, namely commercial food plate Al. The experiment was tailored at a specific target of nanoporous fillers obtained from milled APA to be loaded into nanocomposites for dental restoration, an application demonstrated previously in our group [27]. The criteria identifying the good APA for this application are that the layer thickness be close to the final 3D diameter of the fillers, which should be µm-scale to carry many nanopores yet not too large (<10 µm) to avoid major light scattering; the pore diameter *d* should be large enough for the composite resin to infiltrate it easily (>100 nm), yet the cell diameter be larger enough to provide robust filler structure. As a combination of these requirements, we decided to set the following ranges for the target: pore diameters and spacing of 100–150 nm and 150–275 nm, respectively, and thickness of 6–8 µm.

As the process parameters (also referred to as input factors), we selected the anodization voltage *U*, the electrolyte temperature *T*, and the anodization time *t*. As the main output parameters describing the fabricated anodic porous alumina (APA) (also referred to as response variables), we selected the pore diameter *d*, the cell diameter *D*, and the APA thickness *s*. Other important anodization process parameters could be the electrolyte acid concentration *c* and the total Al foil free surface area *A_Al_* (or, equivalently, the observed current density *j* = *i*/*A_Al_* where *i* is the current passing during anodization, which is also functionally related to *U* and/or *T* and *c* by the laws of electricity and electrochemistry) [28]. In different systems, the type of acid and Al raw material (degree of purity and/or type of alloy) could be considered as well. However, we decided to restrict our study to the selected process parameters because existing literature [1,3] and our experience based on prior experiments [10,27] clearly indicate that *U*, *T* and *t* are the most important factors affecting the process output.

The optimization of APA fabrication by means of statistically designed experiments (DoE) has already been the subject of previous literature [29,30,31,32,33], but in some cases [30,32,33] ultrapure Al was used, while in some other cases [29,31] optimization dealt with ideal target values of the geometrical parameters, disconnected from real applications. Still, another work on optimized nanoporous oxide fabrication was focused on anodization of titanium to increase wettability as a secondary, indirect response [34]. To the best of our knowledge, this work reports the first attempt to optimize APA fabrication starting from consumer quality Al and with the goal to address a specific real application of the nanostructured material resulting from anodization.

## 2. Results

### 2.1. Characterization of the Preliminary APA Dataset

**Figure 1:**
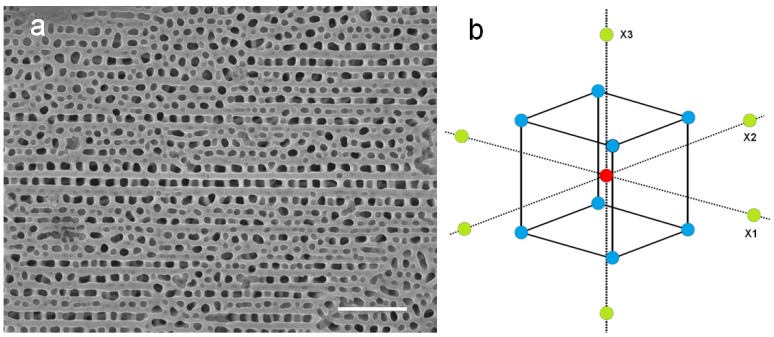
(**a**) Typical raw data image of anodic porous alumina (APA) from food plate (datapoint 1_1) (the scale bar is 1 µm long); (**b**) Sketch of the central composite design (CCD) used in this work, where a central datapoint (in red) has 14 surrounding datapoints (six axial datapoints in green and eight corner datapoints in blue) in the 3D space of a 3-factor statistically designed experiment (DoE). (See correspondence to dataset 2 in Table 2 and Figure S3).

The preliminary APA dataset (dataset 1) available before the start of the present work was properly analyzed by means of scanning electron microscopy (SEM) imaging and subsequent image analysis, as described in the experimental section. In Figure 1a, a typical SEM image of the APA samples is presented in raw data form. The segmentation resulting after grain analysis of the same image is shown in Figure S1. Appearing in Figure 1a is a typical characteristic of the porous pattern of the present APA, obtained from food plate Al, which is the presence of pores mainly aligned along the pre-existing layer on the Al foil. Additionally, some defective areas appear, probably due to contamination or the presence of different metallic elements that are not oxidized during the process [4,28]. 

Actually, since there is no single best threshold that can simultaneously identify both the number of pores and their correct size, two different thresholds were used for these different tasks: one threshold included all the pores but made them too large; another threshold missed some pores (same as in Figure S1, typically 10%) but assigned the correct size to the identified pores (still enough in number, typically 300, to generate good statistics). 

A datapoint in a dataset is indicated with *i*_*j* where *j* = 1, 2 indicates the dataset and *i* the point within the dataset; in particular, the points in the first dataset are indexed with 1_1-6_1 and those in the second batch with 1_2-15_2. It should also be mentioned that each dataset corresponds to a batch of datapoints obtained in a single set of fabrication runs. The input parameters, main responses and secondary responses of dataset 1 are presented in Table S1. For each point in dataset 1, fabricated according to the ‘Operating parameters’ on the left side of Table S1, the SEM image analysis allowed the estimation of the resulting values of the geometrical APA parameters, named ‘Main response’, as well as, consequentially, the ‘Secondary responses’.

### 2.2. DoE Design of the New APA Dataset

From the point of view of the experimental design, the critical operating parameters in Table S1 determining experimental control have been assumed as the factors, whereas the output parameters have been assumed as the responses. The proper form and size of a new dataset, dataset 2, has been designed according to the model in Figure 1b, in order to best probe the correlations between the factors and their consequences on the responses. For the latter, only the primary response parameters (*d*,*D*,*s*) have been considered. Admissible ranges for the control variables are *U* = [50,150] V, *T* = [0,30] °C, and *t* = [1,15] h. Ideally, we would like *s* to be between 6 and 8 μm (with *d* between 100 and 150 nm, and *D* between 150 and 275 nm), and a short time. 

In dataset 2, designed according to the central composite design (CCD) method, in addition to the center datapoint (*U*,*T*,*t*) = (100,15,5), there are six axial datapoints, two along each of the three axes corresponding to the three DoE factors, and eight factorial datapoints, one for each of the eight quadrants in which the three axes split the 3D space of the design factors. The dataset emerging from the CCD method does not need to have points on the surface of the square in Figure 1b; what matters is that the three design factors take five values each. 

Altogether, there are eight variables, three factors (*U*,*T*,*t*) and two sets of response variables, main (*d*,*D*,*s*) and secondary responses (*σ*,*p*). Data used in the analysis have been collected in two batches, corresponding to the two datasets: preliminary dataset, namely dataset 1, and designed dataset, namely dataset 2. Considering both datasets 1 and 2, the selected levels (values) for each factor, in the accessible range, and the number of design points (replicates) for each level are listed in Table 1. For each selected level of each factor (*U*,*T*,*t*), Table 1 gives the number of datapoints with that level, useful for a so-called one-factor at a time analysis, which allows the study of the relationship of response variables and just one design factor. The replicates in each level can be used to estimate the magnitude of the experimental error, which is the experimental variance.

Altogether, considering both available datasets, only the datapoint (110,5,13) is replicated, and both replicates are in dataset 1. The differences in the responses on the replicates of (110,5,13) are not too high: deviations in d and D are small (1% and 2%, respectively), and only deviation in *s* might be significant (~7%).

Table S2 gives the two-factors at a time representation of the points in dataset 1 and dataset 2. Namely, the projection of the points in the two datasets over the three coordinate planes is represented in Table 1 in which the star-shaped structure of the CCD design is visible. The 3D plot of the datasets is given in Figure S3 which also includes the confirmation dataset.

### 2.3. Statistical Analysis of Datasets 1 and 2

The full dataset 2 is in Table 2. On the left side are the input values of the controlled factors (the CCD). On the right side of Table 2, similarly to Table S1, are the observed values (responses) of the APA fabrication and subsequent imaging and image analysis.

**Figure 2:**
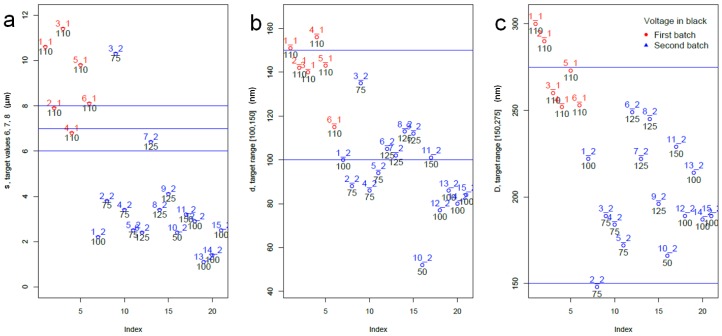
Univariate plots of primary response variables by batch (red for dataset 1 and blue for dataset 2) and voltage (black label); (**a**) APA thickness *s*; (**b**) pore diameter *d*; (**c**) cell diameter *D*.

In Figure 2, where each single main response is considered against the two batches and the voltage variable, a batch effect is evident. Indeed, most points in the first batch of experimental runs corresponding to the preliminary dataset (red circles and labels), have higher *s*, *D* and *d* values than points in the second batch. Furthermore, Figure 2 provides a neat representation of the out-of-target and of the most-in-target datapoints. 

**Figure 3:**
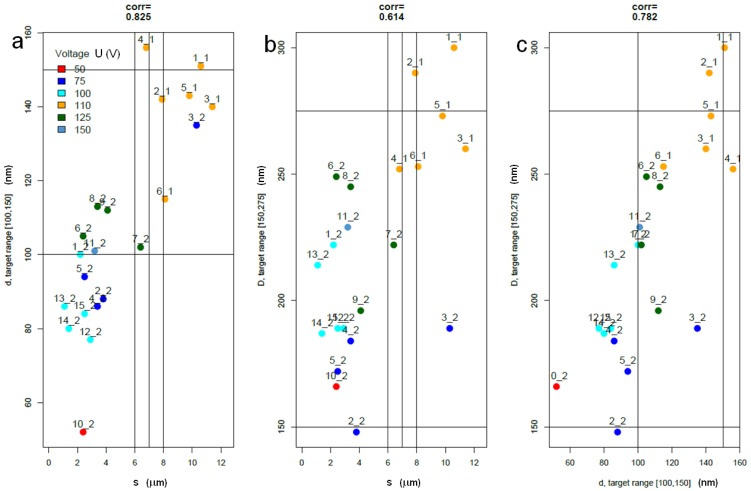
Bivariate scatter plots and correlations; (**a**) *d* versus *s*; (**b**) *D* versus *s*; (**c**) *D* versus *d*. Note datapoints 7_2 and 6_1.

In Figure 3, the bivariate plots of the main response variables appear colored by voltage. In fact, *U* is the only variable among the factors (*U*,*T*,*t*) that gives rise to an apparent clusterization effect. Figure 3 shows that two datapoints are closest to the target, namely 7_2 with (*U*,*T*,*t*) = (125,20,7) and 6_1 with (110,15,5). They are candidates to be optimal datapoints. 

Also included in Figure 3 are the correlations between pairs of main response variables. There is strong observed correlation (~0.83) between *d* and *s*, and weaker (~0.61) between *D* and *s*. The full correlations between pairs of the eight variables in the dataset are in the lower triangle of the matrix in Table 3. The upper triangle of the matrix in Table 3 contains the partial correlation, namely the correlation of any two distinct variables conditional on the remaining variables. 

## 3. Discussion

### 3.1. Previous Literature on the Use of DoE Applied to APA Fabrication

Few works are available in the literature on the DoE analysis of nanoporous alumina fabricated by anodization. In fact, Wang et al. [34] reported on nanopores from anodization in titania, while Barmala et al. [35] reported on the DoE of nanoporous alumina obtained by a different route of sintering assembly of nanoparticles. To the best of our knowledge, the only papers that deal with the application of DoE to APA are [29,30,31,32,33]. Among them, the study by Santos et al. [33] aimed to understand pore rearrangement at the interface between APA layers obtained by mild and hard anodization regimes, and used the two factors of final voltage and ramp rate with three levels each. More similar to our study, Chen et al. [31] used a system with three factors and two levels each, with a dataset consisting of eight runs plus one run of a confirmation experiment. They characterized the success of the confirmation experiment qualitatively, providing information on the deviation from expected values. Their factors were similar to ours, namely (*U*,*T*,*c*) instead of (*U*,*T*,*t*), where *c* was the electrolyte acid concentration. The response variables were the same as ours, (*d*,*D*,*h*). 

Hassanzadeh et al. [32] used the same three factors as Chen: (*U*,*T*,*c*), with three levels each, but only one response, namely *d*. They used an orthogonal L9 design, i.e., a dataset consisting of nine runs, and did not run any confirmation experiment. A similar L9 design was used by Monfared et al. [29], with four factors (*U*,*T*,*t*,*c*); the same as our design with the addition of concentration *c* (the acid was different: oxalic). The output was (*d*,*σ*). No confirmation experiment was run, the same as for [32]. Finally, Nemati et al. [30] used as the factors (*t*,*j*), with *j* anodization current density (three levels each in a full-factorial design), and as the output the sensitivity of an APA-based molecular sensor working by means of interference reflectance spectroscopy. 

### 3.2. General Considerations and Limitations of Present Work

It should be mentioned that the values for the ‘Operating parameters’ are exact, within the experimental accuracy of the setting controls (relative error <1%). For the ‘output results’, instead, where possible, we took into account the uncertainty in the respective quantities. More precisely, the values for *σ* and *p* were approximated to the closest integer, whereas for *s* a single digit after the decimal separator was considered to be significant. For the values of *d* and *D*, instead, the mean values of all the pores in the given image (with *N* in the range of 200–400) were reported. In fact, these values are affected by different sources of uncertainty. First, the SEM resolution itself can be estimated at around 10 nm. Additionally, an uncertainty of typically ~30 nm and ~50 nm can be ascribed to *d* and *D* respectively, as resulting from the respective standard deviations of the pore populations. Nevertheless, while we did not want to overestimate the measurement accuracy, we still assumed that these means are the most correct representation of the *d* and *D*, without performing any further approximation. 

Concerning the nature of the starting Al used, we have performed SEM measurements with additional energy dispersive spectroscopy (EDS) to assess its type (see Figure S4); information which was not provided by the manufacturer. While the purity was relatively high (~97.4 wt % Al), identification of the alloy was unclear, the major alloying element being Fe (~0.8 wt %, for details see Table S4). It should be mentioned that it is not easy to foresee and model the effect of the alloy impurities; this is the subject of a book currently in press [36]. Generally speaking, it is expected that the impurities pass through the Al anodization intact, due to their lower anodization potential (except Mg, when present, whose oxidation potential of −2.38 V is lower than the −1.66 V of Al). However, the impurities represent an electrical resistance in the circuit, giving rise to parasitic effects (Joule heating with possible burning, or even preventing the anodization, which was fortunately not the case here). The metal cations, different from Al^3+^, may accumulate at the oxide interfaces (including the knitlines) and provide APA coloring effects. However, a detailed assessment of their behavior and consequences on the APA morphology to composition or microstructure is beyond the scope of this work.

### 3.3. Discussion of Present Results

Since porosity is the coverage of pores with respect to the projected surface area and pore density is the numbers of pores per unit area, it is expected that *p* = (*d*/*D*)^2^ and *σ* = 4/*πD^2^*. These expected relationships have been verified by plotting the respective calculated quantities, (*d*/*D*)^2^ and 4/π*D*^2^, against the measured values of *p* and *σ*, for all the datapoints in datasets 1 and 2 and also the confirmation experiment (see Figure S2). 

The theoretical relationship for *p* = (*d*/*D*)^2^ is satisfied by our datasets as shown in Figure S2a, with the exception of the datapoint 10_2. Also, the relationship *σ* = 4/(*πD*^2^) is satisfied by our datasets, as shown in Figure 2b. The datapoints 3_2 and 2_2, while still satisfying the expected relationships, are, nonetheless, outliers in the two plots in Figure S2. It can be observed that both are characterized by low values of the voltage parameter, which could be a hint of the anodization process instability when running at low voltages. Furthermore, as datapoint 10_2 has the lowest *s* (2.4 µm) and *d* (52 nm) values, which are outside the target region, we could safely exclude it from the analysis. 

**Figure 4:**
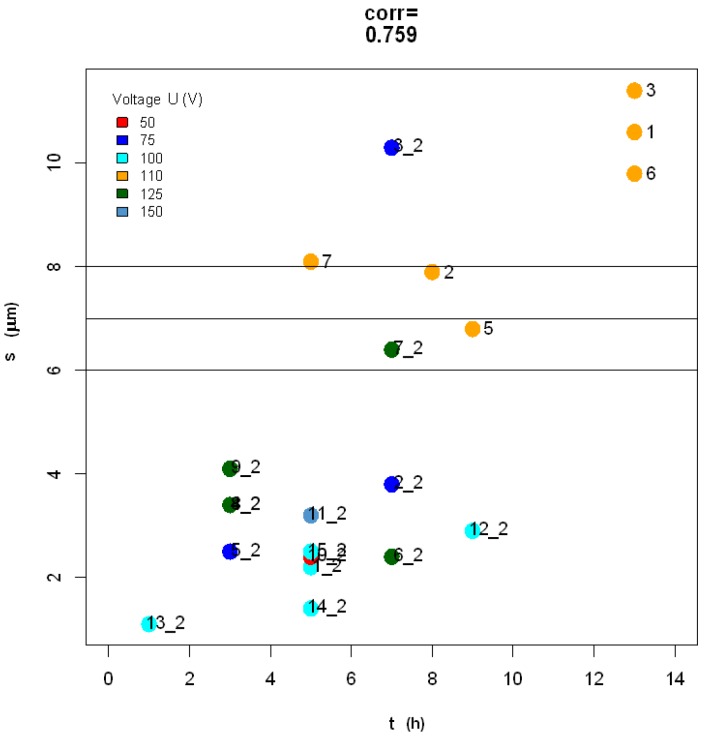
APA thickness *s* versus anodization time *t*: the datapoints are largely scattered along the diagonal straight line in the plot.

On the basis of previous knowledge, the APA thickness *s* should be roughly proportional to the anodization time *t*. [1,10] However, it should be pointed out that the experimental anodizations in Reference [10] were carried out in galvanostatic mode. Our present data do not clearly support the assumption of proportionality between *s* and *t*, as shown in Figure 4. 

**Figure 5:**
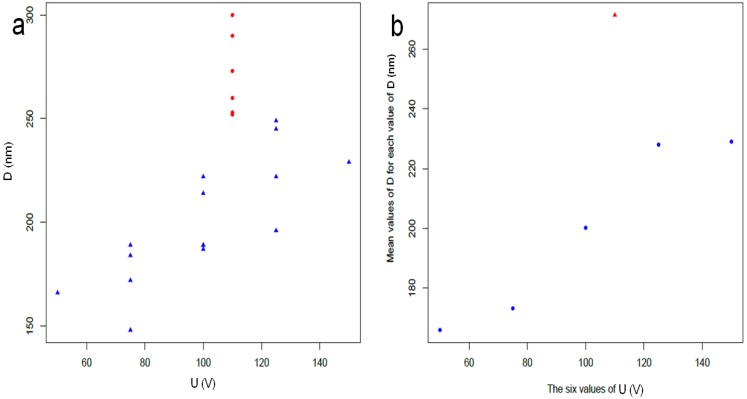
(**a**) *D* versus *U* for all datapoints; (**b**) *D* versus *U* points after averaging the *D* values corresponding to the same *U* values. The apparent lack of linearity between *D* and *U* is mainly imputable to the batch effect, as shown by the red dots indicating points in dataset 1.

Similarly, the correlation between *D* and *U* is only 0.61 while their partial correlation is 0.21 (see Table 3). This does not support the linearity between *D* and *U* as confirmed by the two plots in Figure 5. This would be against long-standing knowledge emerging from most works on APA [1,3]. However, it should be observed that deviations are due to the so-called batch effect. Indeed, the linearity in Figure 5 is mainly missing due to the red dots, belonging to dataset 1. This is especially clear when the datapoints at the same *U* value are averaged, as shown in Figure 5b. 

In conclusion, there appears to be a notable batch effect. The datapoints in dataset 1, corresponding to the first batch, have *s* closer to the target than most datapoints of dataset 2, corresponding to the second batch. *d* and *D* are mostly within the target and thus the focus of further analysis should be on *s*. When only dataset 1 was available, datapoint 6_1, which was almost on target with respect to *s* and had low *t*, seemed to be optimal. Over the two batches, the most on-target datapoints with a reasonable small value of *t* are 6_1 and 7_2 (see Tables S1 and S2 for the values of factors as well as responses). 

### 3.4. Confirmation Experiment

To confirm whether the candidate datapoints are optimal, as mentioned above, we decided to finally run a confirmation experiment, consisting of five new runs. These new datapoints have filled in what we called dataset 3, see Table S3. A 3D plot of all the design points considered in this paper is given in Figure S3 with respect to the three design factors. 

Points 4_3 and 5_3 were planned for *t* = 9 h and 5 h respectively, but the process was stopped earlier due to logistic reasons unrelated to the process itself. The type of statistical analysis we performed in this paper and its conclusion are still valid even if the planned confirmatory experiment was a regular CCD while the actually performed experiment did not exhibit the same geometric regularity. Overall, we see from the Main response columns in Table S3 that most datapoints of dataset (and batch) 3 are close to the target values, and only 5_3, which had shortest *t*, deviates significantly from the desired *s* range (6–8 µm). This observation extends to also include point 4_3 but not point 5_3 (for which *s* is well off target). This note about 5_3 supplies evidence to support the conclusion that simultaneous setting of low values of *t* and *T* does not bring optimal main responses. In conclusion, we can consider that the confirmation experiment was successful in supporting the results of dataset 2. 

Finally, in Figure S3, all three datasets are represented in the space of the design factors. Dataset 1 was randomly distributed, dataset 2 corresponds to the implementation of the CCD design shown in Figure 1b, while dataset 3 was designed for confirming the optimal design region suggested by the first two datasets. For the batch effect appearing in dataset1 as compared to dataset 2, *a posteriori* it is difficult to fully understand the experimental reasons for the observed differences. However, it should be mentioned that the preliminary dataset (dataset 1) was collected several months earlier, and by using a former (smaller) version of the anodization cell, which may have introduced some deviations. The present work also aims to show the capability of a detailed statistical analysis to point out possible issues in the experiments. 

## 4. Materials and Methods

### 4.1. APA Fabrication

For the raw Al to be anodized we used commercial food plates found at a shopping village supermarket, model V2 23040501 1200cc (Cuki Cofresco, Volpiano-TO, Italy). The foils of these plates have a center flat ellipsoidal region with orthogonal axes of 15 and 30 cm and ~200 μm thickness. The external borders to the central ellipse are tilted at an angle, to give edges to the shape of the plate, by means of high spatial frequency folding which also reinforces the structure. This external uneven part was cut off, and the remaining flat Al foil was cut in pieces of 5 cm × 8 cm and degreased by wiping both sides with tissue paper soaked in acetone first, then ethanol and finally water. As the last step of the preparation process, the surface was blown dry with nitrogen. Anodization was carried out in 0.4 M phosphoric acid (H_3_PO_4_, Sigma, Milan, Italy). The counter-electrode, namely the cathode (set to ground voltage, i.e., negatively biased with respect to the positive Al anode) was a ~1 mm thick platinum wire, curled to form a spiral with distance between adjacent wire segments of 3 mm and outermost diameter of 5 cm. This cathode was placed in front of the Al anode, which was dipped in the electrolyte along the long side to a depth of approximately 5 cm. The uppermost 3 cm were used to create a physical contact with a crocodile clamp, making the distance from the solution meniscus long enough to ensure that the clamp never touched the electrolyte. Stirring was applied by means of a Teflon-coated magnet on the bottom of the double-walled (jacketed) beaker, at a distance of 5 cm from the bottom-most edge of the Al foil. The system was cooled by means of a thermocryostat Pro-line RP 3530 C (Lauda, Lauda-Königshofen, Germany), filled with refrigerating liquid Kryo 40 from the same company.

All chemicals were from Sigma and were used as received. For water, we mean deionized water, as obtained from an Elix5 system (Millipore, Vimodrone-MI, Italy).

### 4.2. SEM Imaging and Image Analysis

For evaluation of the geometric figures describing the APA, which determine the response of Al anodization, we used SEM imaging. A microscope model JSM 649OLA (JEOL, Tokyo, Japan) was used, at acceleration voltage of 15 kV and standard magnification of 20,000x. On each specimen, at least three different regions were inspected, to check the uniformity of surface patterning into pores obtained by anodization. The images were 8-bit gray levels, with digital size of 1280 × 960 pixel, and a scale of 4.6729 nm/√pixel. When the three images of the same specimen looked to have similar properties, a single one was randomly taken as representative of the specimen, which was subjected to image processing and analysis within the grain analysis tools of Igor 6.22 (Wavemetrics, Lake Oswego, OR, USA). Without any pre-treatment of spatial low-pass frequency kernel filtering (such as used in different works) [37,38], a threshold level within the range of 256 gray levels was chosen by the expert operator to segment the pores (negative grains) with respect to the background (top of the APA cell walls). The pores crossing the image edges were excluded from identification, as well as the pores too small to be identified as real pores (typical minimum size of 16 pixels), but which can be ascribed to noise in the SEM images or are different types of surface defects in APA. Then, the number of pores *N* was counted, which allowed us to determine the pore density *σ*, according to the relationships *σ* = *N/A* where *A* is the total image area. The porosity was also evaluated, as the coverage of the total area of the pores as compared to the image area, *p* = *NA_p_/A*, where *A_p_* is the mean area of a single pore. From *A_p_*, the pore diameter *d* was evaluated, by assuming circular pores, as *d* = √(4*A_p_*/π). Analogously, the cell diameter *D* was obtained—which is the same as the pore center-to-center spacing—as *D* = √(4*A_c_*/π), where *A_c_* is the cell area, which in turn emerged from *A_c_ = A/N* because the number of cells is the same as the number of pores *N* (one cell–one pore). 

In addition to the top view imaging used for characterization of the pores, SEM was also used on cross sections of fractured APA to assess the respective thickness. Whereas SEM at given magnification would allow, in principle, for much higher accuracy, the difficulty in exact distinction of the Al and the overcoating oxide made us assume an uncertainty in thickness values of ~0.1 µm.

The same SEM has also been used for EDS assessment of the elemental composition of the starting raw material Al used for anodization.

### 4.3. DoE Method

The APA fabrication process parameters identified as of critical importance, namely the anodization voltage *U*, the anodization time *t*, and the electrolyte temperature *T*, were used as DoE input factors. The geometric APA parameters resulting from the anodization, also identified as of critical importance for the considered application of the nanomaterials, namely the pore diameter *d*, the cell diameter *D* and the APA thickness *s*, were used as DoE response (or output) variables. The latter one, *s*, is specifically correlated with the peculiar application of the APA addressed in this work, which requires milling of the APA membrane into micro-particles to be used as the filler in resin composites. In addition to *d*, *D* and *s*, identified as the main response variables, pore density *σ* and porosity *p* were also considered. They are called here the ‘secondary’ response as they are functions of the primary variables. 

The starting available knowledge on the working process was contained in a preliminary dataset, reported in Table S1 and referred to as dataset 1. The samples described in dataset 1 were made before taking the decision to perform the present work, and were analyzed in order to take advantage of the information they contained in view of the design of a new dataset. Unfortunately, this preliminary dataset only contained experimental runs with a single value of *U*, namely *U* = 110 V. After analyzing dataset 1, a CCD in 3D was chosen (see Figure 1b) with a central point similar to point 6_1 of dataset 1, which allowed the definition of a new design dataset (dataset 2). In particular, we are interested in the study of the simultaneous effect on the response variables of varying all design factors. A full factorial design could be employed for that aim but it would require $R_{FF}=\overset{F}{\underset{1}{\prod }}L_{i}$ runs, where *F* = 3 is the number of factors and *L_i_* is the number of levels for factor *i*. This gives a total of 6 × 6 × 7 = 252 runs for a full factorial design. However, our design can be successfully employed for exploring the same space of input variables, with a total number of runs limited to 6 + 15 = 21, including also the preliminary dataset 1. 

CCDs are commonly used in experiments designed for the study of the relationship between the response and the input factors [39] when non-linear relationships are expected. A CCD includes an embedded factorial (or fractional factorial design) with a center point, which can be replicated, and a group of ‘star points’ that allows the estimation of curvature and second order effects. For the three input factors (*U*,*T*,*t*), the selected CCD is formed by a center point (100,15,5), eight corner points (the factorial part) and six axial points, identified in Table 1 with labels 1_2-1_9 and 1_10-1_15, respectively. The distances of the corner points from the center point and of the axial points by the cube face of the full factorial were chosen following expert authors’ advice. Both the statistical analysis and the DoE were implemented in the freeware software R version 3.2.4 (10 March 2016) [40].

## 5. Conclusions

In summary, it has been shown that:

1. Our goal of fabricating comparatively good quality nanoporous alumina (i.e., controlled mean pore size and spacing as well as oxide thickness) for future large-scale production from inexpensive raw material, instead of ultrapure aluminum as currently used in academic research, is feasible. This will pave the way for real applications of this nanostructured material, for example in advanced composites for dentistry, aerospace and automotive uses, thanks to the mechanical interlocking, allowing the removal of the bonding agent phase, as well as in chemical and bio-sensors and in catalysis.
2. In particular, we aimed at reaching target pore size and spacing of 100–150 nm and 150–275 nm for pore infiltration by the resin, and target thickness of 6–8 µm, best for subsequent ball-milling of the peeled-off alumina membranes to be used as the inorganic filler of dental composites, according to previously established procedures, in the shortest possible time. Based on the scanning electron microscopy imaging and the subsequent statistical analysis, the working parameters satisfying these requirements have been identified as 125 V, 20 °C and 7 h.
3. We can partly confirm the expected behavior between operating parameters and emerging geometry, within the limitations emerging due to a batch effect, pointing out the issue or experimental repeatability in the process.
4. The successful run of a confirmation experiment pointed out the possible implementation of an ‘industrial’ process of APA membrane fabrication for the identified application, when all processing parameters are carefully controlled. Most of the plots, which were used in order to determine an optimal design region, can be easily adapted to control the industrial process.

## Figures and Tables

**Table 1:** Selected levels (values) for each factor, in the accessible range, and number of design points (replicates) for each level, for both datasets.

Factor	Level No.	1	2	3	4	5	6	7
*U*	Level	50	75	100	110	125	150	-
	Replicates	1	4	5	6	4	1	-
*T*	Level	5	8	10	15	20	25	-
	Replicates	4	2	4	6	4	1	-
*t*	Level	1	3	5	7	8	9	13
	Replicates	1	4	6	4	1	2	3

**Table 2:** Dataset 2: from left to right, the columns give the identifier of the design point, CCD design, main and secondary responses (see also Figure 1b and Figure S3).

Datapoint		Factors			Main Responses			Secondary Responses	
ID	Type	*U* (V)	*T* (°C)	*t* (h)	*d* (nm)	*D* (nm)	*s* (μm)	*σ* (μm^−2^)	*p* (%)
1_2	Central	100	15	5	100	222	2.2	26	20
2_2	Corner	75	10	7	88	148	3.8	58	35
3_2		75	20	7	135	189	10.3	32	53
4_2		75	10	3	86	184	3.4	37	22
5_2		75	20	3	94	172	2.5	43	30
6_2		125	10	7	105	249	2.4	21	18
7_2		125	20	7	102	222	6.4	26	21
8_2		125	10	3	113	245	3.4	21	21
9_2		125	20	3	112	196	4.1	33	33
10_2	Axial	50	15	5	52	166	2.4	28	9
11_2		150	15	5	101	229	3.2	24	19
12_2		100	15	9	77	189	2.9	36	17
13_2		100	15	1	86	214	1.1	28	16
14_2		100	25	5	80	187	1.4	37	18
15_2		100	5	5	84	189	2.5	36	20

**Table 3:** Marginal correlations in the lower triangle and partial correlations in the upper triangle. The gray background for the cells along the diagonal serves as a guide to the eye. The secondary response cells are also painted on a gray background, as a less important (derived) type of correlation among variables. The colors point out the highest values in absolute value, above 70% (red) and above 80% (blue).

	*T*	*t*	*U*	*s*	*d*	*D*	*p*	*σ*
***T***	1.00	−0.23	0.29	0.17	−0.25	0.06	0.23	−0.18
***t***	−0.52	1.00	0.05	0.64	0.23	−0.16	−0.31	0.26
***U***	−0.12	0.14	1.00	−0.35	0.03	0.21	0.02	0.11
***s***	−0.37	0.76	0.12	1.00	−0.10	0.19	0.29	−0.25
***d***	−0.45	0.63	0.41	0.83	1.00	0.90	0.96	−0.04
***D***	−0.51	0.56	0.61	0.61	0.78	1.00	−0.87	−0.29
***p***	−0.01	0.24	−0.12	0.54	0.59	−0.03	1.00	0.14
***σ***	0.37	−0.42	−0.53	−0.51	−0.62	−0.91	0.17	1.00

## Supplementary Materials

The following are available online at www.mdpi.com/1996-1944/10/4/417/s1: document including Figures S1–S4, Tables S1–S4.
