# Supplementary material for: Optimization of Anodic Porous Alumina Fabricated from Commercial Aluminum Food Foils: A Statistical Approach
Source: Materials (Basel). 2017 Apr 15;10(4):417. doi: 10.3390/ma10040417 (PMC5506948; doi:10.3390/ma10040417)
Supplement: Supplementary file 1 [file materials-10-00417-s001.pdf]

Supplementary Materials: Optimization of Anodic Porous Alumina Fabricated from Commercial Aluminum Food Foils: A Statistical Approach

Eva Riccomagno, Amirreza Shayganpour and Marco Salerno

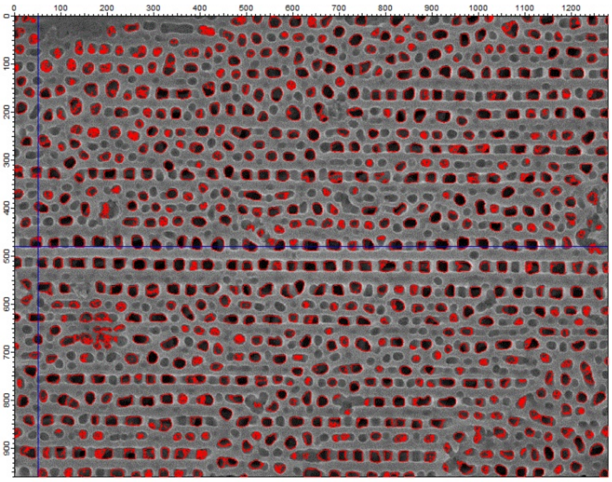

Figure S1. The same image as in Figure 1a (with side numbers representing pixels) after pre-treatment and grain (pores) analysis, with threshold set for best pore size assignment.

Table S1. Preliminary dataset (shortly dataset 1). Each row refers to a datapoint. The first column shows the index of the datapoint, the next three columns give the input parameters, the central columns the main responses and the last two columns secondary responses. Replicates are indicated using gray cell background.

	Input Parameters			Main Responses			Secondary Responses	
Data Identifier	U (V)	T (°C)	t (h)	d (nm)	D (nm)	s (μm)	σ (μm^{-2})	p (%)
1_1	110	8	13	151	300	10.6	14	25
2_1	110	8	8	142	290	7.9	15	24
3_1	110	5	13	140	260	11.4	19	29
4_1	110	5	9	156	252	6.8	20	38
5_1	110	5	13	143	273	9.8	17	27
6_1	110	15	5	115	253	8.1	20	21

Table S2. 2D representation of the design with replicates for dataset 1 and dataset 2.

Tt	5	8	10	15	20	25
1				1		
3			2		2	
5	1			4		1
7			2		2	
8		1				
9	1			1		
13	2	1				

Ut	5	7	100	11	12	15
1			1			
3		2			2	
5	1		3	1		1
7		2			2	
8				1		
9			1	1		
13				3		

TU	5	8	10	15	20	25
50				1		
75			2		2	
100	1			3		1
110	3	2		1		
125			2		2	
150				1		
150				1		

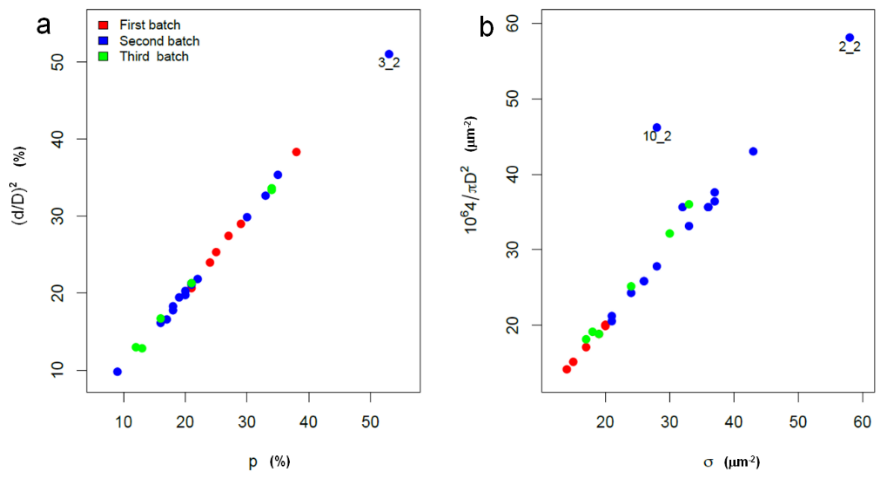

Figure S2. (a) $(d/D)^2$ versus p and (b) $4/(\pi D^2)$ versus σ, by voltage.

Table S3. Confirmation dataset, shortly called dataset 3, including the results of its analysis.

	Factors			Main Responses		Secondary Responses		
Datapoint	U (V)	T (°C)	t (h)	d (nm)	D (nm)	s (µm)	σ (µm^{-2})	p (%)
1_3	100	20	9	92	225	8.8	24	16
2_3	100	8	5	115	199	6.4	30	34
3_3	110	15	8	120	260	8.7	19	21
4_3	120	20	5	93	258	6.5	18	12
5_3	120	8	4	95	265	2.8	17	13

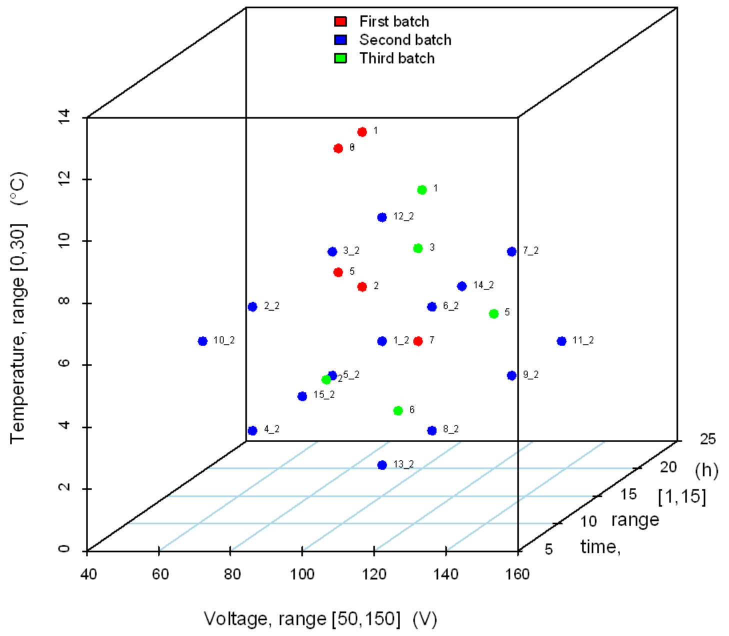

Figure S3. 3D plot of the three datasets (and batches) in the space of design factors. (See correspondence of dataset 2—in blue—to Figure 1b).

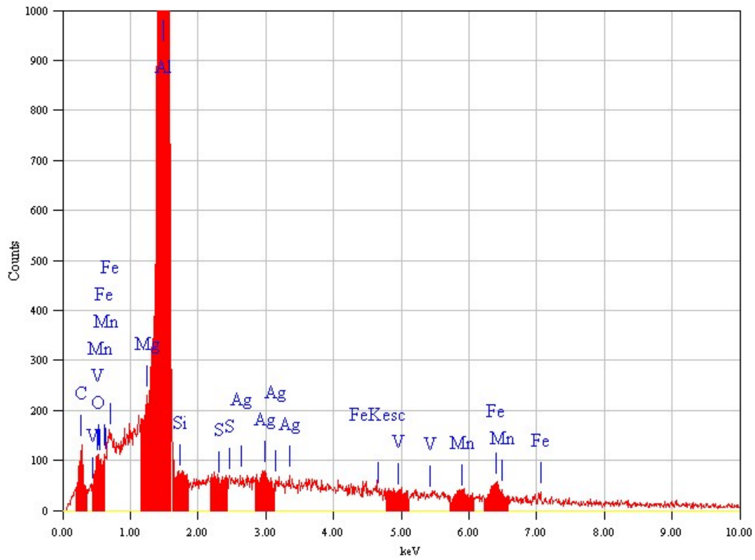

Figure S4. EDS spectrum of the starting Al anodized during this work, with assignment of the identified elements present in the sample.

Table S4. Quantitative analysis for the main elements identified in the spectrum of Figure S1.

Element	Line Type	Line Energy (keV)	Mass (%)	Error (%)	Atom (%)
Al	K	1.486	97.44	0	97.43
Fe	K	6.398	0.8	0.88	0.39
C	K	0.277	0.68	0.09	1.54
Mn	K	5.894	0.38	1.76	0.19
Ag	L	2.984	0.47	1.56	0.12
O	K	0.525	0.19	0.41	0.32
V	K	4.949	0.03	14.2	0.02
